# Factors associated with long-term survival after traumatic and non-traumatic spinal cord injury: a 12-year population-based retrospective cohort study in Italy

**DOI:** 10.1038/s41393-025-01162-1

**Published:** 2025-12-23

**Authors:** Alberto Borraccino, Roberta Onorati, Alessio Conti, Andrea Ricotti, Carlo Mamo

**Affiliations:** 1 Department of Clinical and Biological Sciences, University of Torino, 10043 Orbassano (TO), Italy; 2 Epidemiology Unit, Local Health Authority TO3, 10093 Collegno, Italy; 3 Clinical Trial Unit, AO Ordine Mauriziano Hospital, Turin, 10128 Italy

**Keywords:** Risk factors, Epidemiology, Outcomes research

## Abstract

**Study Design:**

retrospective, population-based cohort study.

**Objectives:**

to evaluate long-term survival outcomes and factors affecting mortality in individuals with traumatic (TSCI) and non-traumatic spinal cord injury (NTSCI) in Italy.

**Setting:**

Publicly funded rehabilitation units for SCI patients across Italy.

**Methods:**

A total of 1070 individuals with confirmed TSCI (56.5%) or NTSCI, predominantly male (70.5%), residing in the Piedmont region, were identified through admission to specialized SCI rehabilitation units (2008–2020) using administrative data. Survival probabilities were estimated using Kaplan-Meier curves; Mortality risk was assessed using multivariate Cox regression models, and adjusted hazard ratios (HRs) were reported for sex, comorbidity burden (combined Charlson Comorbidity Index, CCI), and injury level (paraplegia/tetraplegia). Causes of death were retrieved from national mortality records.

**Results:**

The overall case fatality rate was 25.1%, higher in NTSCI (32.9%) than in TSCI (19.2%). Mortality increased with age and comorbidity burden. Among individuals with TSCI and CCI ≥ 3, mortality risk was significantly higher (HR 1.81; 95% CI 1.29–2.53). Sex (HR 1.05) and injury level (HR 1.10) were not significant predictors. Leading causes of death were cancer in NTSCI and circulatory diseases in both groups.

**Conclusions:**

Age, comorbidities and injury type are the primary determinants of survival in SCI. The higher case fatality rate observed in NTSCI compared with TSCI underscores the prognostic relevance of etiology alongside frailty and multimorbidity. Our findings support early frailty screening, personalized comorbidity management, and the need for developing a dedicated national registry to inform long-term care strategies and improve outcomes in aging SCI populations.

## Introduction

Spinal cord injury (SCI) is a life-altering condition that can arise from traumatic (TSCI) or non-traumatic (NTSCI) causes, resulting in significant consequences for individuals, their families and health systems. TSCIs are commonly associated to road traffic, home and work accidents or sports injuries, whereas NTSCIs frequently occur as a result of underlying pathologies, including cancer, vascular disorders or infections [1]. Both forms give rise to variable degrees of neurological dysfunction, creating a significant demand for specialized long-term care and rehabilitation [2, 3].

Globally, SCI epidemiology is shifting. While the incidence of TSCI has shown relative decline in some regions due to preventive efforts targeting road and workplace accidents, NTSCI rates are rising, largely in older adults with multiple comorbidities. This trend has been accompanied by increasing age at injury onset (from 55 to 65 years), a narrowing male-to-female ratio, and greater complexity in clinical management due to the presence of chronic conditions. Consequently, the burden of SCI is evolving, with long-term care increasingly shaped by age-related frailty and multimorbidity [4, 5]. These factors, alongside advancements in preventive strategies and improvements in available healthcare technologies, have contributed to amplifying the complexities and economic burden surrounding this condition, thereby increasing the direct and indirect costs associated with SCI management [6].

Despite significant efforts to improve rehabilitation strategies and healthcare infrastructure, there is wide variability in survival estimates and mortality patterns among SCI populations, especially between TSCI and NTSCI groups. Reported life expectancy after SCI ranges broadly (between 2.3 and 43 years) [7], and mortality risks are consistently elevated when compared to the general population (from 1.62 for NTSCI to 2.45 for TSCI) [8]. However, longitudinal studies investigating how pre-existing comorbidities affect SCI prognosis remain limited. Most research has focused on short-term outcomes (e.g., in-hospital mortality), or on selected subpopulations, without distinguishing between injury etiologies [9] or stratifying for comorbidity burden [10, 11].

Moreover, existing literature is often characterized by methodological heterogeneity [9, 12], regional bias, or small sample sizes, limiting generalizability [13, 14]. There is a growing consensus on the need for comprehensive, population-based analyses that can disentangle the influence of clinical and demographic factors, particularly comorbidities, on long-term outcomes. This is especially relevant for NTSCI, where underlying conditions, such as cancer or vascular diseases, often drive prognosis more than the spinal injury itself [15].

In Italy, epidemiological data on SCI remain scarce and fragmented. Few studies have offered detailed analyses of long-term survival, and even fewer have leveraged the full potential of administrative databases to capture comorbidity profiles, healthcare trajectories, and cause-specific mortality. The use of official national data sources, such as the Hospital Discharge Records alongside with the Mortality Information System, offers a valuable opportunity to generate robust, generalizable evidence across diverse settings and populations. Administrative databases allow for comprehensive, long-term follow-up and accurate linkage between rehabilitation episodes, comorbidity indices, and mortality outcomes. Their integration can support data-driven decision-making at clinical and policy levels by identifying risk factors amenable to early intervention and informing resource allocation strategies in SCI care [16, 17].

This population-based cohort study aims to evaluate long-term survival and mortality outcomes in individuals with traumatic and non-traumatic SCI in investigating whether these outcomes can be influenced by factors such as age, sex, injury level and pre-existing comorbidities. Through the analysis of 12 years of ministerial administrative data, the study seeks to quantify the impact of these variables on survival trajectories, with the objective of generating evidence to guide targeted interventions and optimize care planning for the SCI population.

## Methods

This retrospective cohort study included all residents of the Piedmont region (northwestern Italy) who were first admitted to a public specialized rehabilitation unit following a SCI between 1 January 2008 and 31 December 2020 in Italy. Only individuals who survived the acute phase, covering initial hospitalization, intensive care, and any necessary surgical management were included. Given the completeness of national administrative databases, all eligible cases were captured, as only survivors could be recorded in the rehabilitation discharge data.

### Setting and study sample

Italy has a universal public healthcare system providing comprehensive medical services, including general practice, hospital care, long-term care, and rehabilitation at no direct cost to patients. The country hosts a widespread network of specialized SCI rehabilitation centers. Piedmont, the second-largest Italian region (over 25,000 square kilometers) had about 4.3 million residents in 2020, ranking seventh in population size.

### Inclusion and exclusion criteria

Eligible participants:

i) Residents of Piedmont at the time of injury;
ii) aged ≥ 15 years at the time of admission in the rehabilitation unit;
iii) First admission to a public specialized SCI rehabilitation unit in Italy between 1 January 2008 and 31 December 2020 and no prior SCI diagnosis;
iv) Confirmed diagnosis of TSCI or NTSCI, established prior to admission to a rehabilitation unit. Injury type was identified through ICD-9-CM codes: TSCI included vertebral fractures with SCI (806.0–806.9) and SCI without spinal bone injury (952.0–952.9), while NTSCI included diagnoses such as spinal neoplasms, vascular myelopathies, infectious or inflammatory myelitis, and other specified cord pathologies (e.g., 192.2, 198.3, 225.3, 323.x, 336.1 and 336.9), spinal stenosis other than cervical (724.0 to 724.09); tuberculoma of the spinal cord (013.4); tuberculous abscess of the spinal cord (013.5); and tuberculous encephalitis or myelitis (013.6 to 013.66); 724.0x, 013.4–013.66);

Data were drawn from the Hospital Discharge Form database, where diagnoses are coded by clinicians at discharge. ICD-9-CM codes were not used to categorize injury type (traumatic vs. non-traumatic) among clinically diagnosed rehabilitation admissions [2].

Individuals who died during the acute phase or had vertebral fractures without SCI, isolated nerve root injuries, or other neurological conditions not meeting SCI diagnostic criteria were excluded.

### Data collection and follow-up

All eligible subjects were followed from admission to death or study end. Demographic data were extracted from the Italian Census Database and linked with mortality records from the National Information System of Mortality. Comorbidities were assessed using the combined Charlson Comorbidity Index (CCI), calculated from hospital discharge records in the 12 months before SCI. The index assigns weighted scores to selected chronic conditions based on their impact on mortality [10] and adds one additional point for every decade of age over 50 years (e.g., 1 added point for ages 50–59, 2 points for 60–69, and so on), reflecting overall health status. The combined CCI is a validated tool developed and adapted by Charlson et al. [18], and has been widely used to assess the frailty of SCI survivors [11],

Diagnostic data were extracted from the national Hospital Discharge database using ICD-9-CM codes. Neurological status was assessed and reported according to the ASIA/ISCoS International Standards for Neurological Classification of Spinal Cord Injury (ISNCSCI).

### Mortality and cause of death assessment

Causes of death were obtained from the Italian Information System of Mortality (ISM), managed by the National Institute of Statistics (ISTAT) (https://www.istat.it/en/data/databases). The system records mortality data using ICD-10 codes. Due to national data availability, cause-of-death information was accessible for the cohort only up to 2018.

### Statistical analyses

Mortality rates were calculated as deaths per 1000 person-years of follow-up, stratified by sex, age group, injury level, CCI score, and rehabilitation center. Associations between all categorical variables were tested using the Cramér’s V. Case fatality rate was expressed as a percentage of deaths among individuals diagnosed with SCI during the study period.

Survival Analysis were reported through Kaplan-Meier survival curves. Plots were generated to estimate the cumulative survival probability over time, stratified by TSCI and NTSCI, as well as by sex and injury level. Group differences in survival were assessed using the log-rank test and the Breslow test.

Multivariate Cox proportional hazards regression models were used to estimate the risk of mortality and expressed as hazard ratios (HRs) with 95% confidence intervals (CIs). The models included sex, injury level, and CCI score as independent variables. To account for center-level heterogeneity, all multivariable models included fixed effects for the SCI rehabilitation unit. All models were adjusted for sex, and SCI rehabilitation unit; age at SCI diagnosis and pre-existing comorbidities were assessed using the combined CCI score.

Statistical analyses were performed using SAS version 9.4 (SAS Institute, Cary, NC, USA).

This study is reported in accordance with the STROBE statements [19].

## Results

### Study population

This retrospective cohort study included 1070 residents of the Piedmont region admitted to a public rehabilitation unit in Italy between 2008 and 2020 (Table 1). Of these, 605 (56.5%) had a TSCI and 465 (43.5%) a NTSCI, accounting for 6619 person-years of follow-up. Overall, 70.5% of the cohort were male.

**Table 1:** Demographic and clinical characteristics of individuals with SCI, stratified by etiology (traumatic vs. non-traumatic), subjects alive in December 2020 and deaths that occurred between 2008 and 2020.

	TSCI			NTSCI			Overall	
	subjects alive in December 2020	deaths between 2008 and 2020	all	subjects alive in December 2020	deaths between 2008 and 2020	all		
	(n = 489)	(n = 116)		(n = 312)	(n = 153)		Fatality rate	Person-year
	n (%)	n (%)	N	n (%)	n (%)	N	%	1000 p.y.
**Gende**r^†^								
males	393 (80.4)	86 (74.1)	479	179 (57.4)	97 (63.4)	276	24.2	40.5
females	96 (19.6)	30 (25.9)	126	133 (42.6)	56 (36.6)	189	27.3	51.1
**Age class**^†^								
15 to 29 years of age	106 (21.7)	2 (1.7)	108	28 (9.0)	3 (2.0)	31	3.6	5.2
30 to 44 years of age	140 (28.6)	7 (6.0)	147	55 (17.6)	7 (4.6)	62	6.7	8.6
45 to 59 years of age	137 (28.0)	24 (20.7)	161	88 (28.2)	30 (19.6)	118	19.3	32.0
60 to 74 years of age	82 (16.8)	46 (39.7)	128	109 (34.9)	79 (51.6)	188	39.6	86.4
over 75 years of age	24 (4.9)	37 (31.9)	61	32 (10.3)	34 (22.2)	66	55.9	182.5
**Impairment**^†^								
paraplegia	275 (56.2)	49 (42.2)	324	210 (67.3)	114 (74.5)	324	25.1	42.0
tetraplegia	199 (40.7)	65 (56.0)	264	75 (24.0)	31 (20.3)	106	25.9	50.0
**Charlson Comorbidity index**^†^								
zero	408 (83.4)	76 (65.5)	484	203 (65.1)	80 (52.3)	283	20.3	37.0
Between 1 and 2	67 (13.7)	17 (14.7)	84	68 (21.8)	33 (21.6)	101	27.0	36.4
Equal 3 or higher	14 (2.9)	23 (19.8)	37	41 (13.1)	40 (26.1)	81	53.4	103.8
**Rehabilitation department**^†^								
SCI unit in Torino	270 (55.2)	68 (58.6)	338	157 (50.3)	69 (45.1)	226	24.3	41.1
SCI unit in Alessandria	84 (17.2)	22 (19.0)	106	51 (16.3)	45 (29.4)	96	33.2	64.0
SCI unit in Novara	77 (15.7)	16 (13.8)	93	64 (20.5)	31 (20.3)	95	25.0	49.9
SCI units outside region	58 (11.9)	10 (8.6)	68	40 (12.8)	8 (5.2)	48	15.5	20.6

Effect size estimates were tested using Cramér’s V^†^.

Missing in level of injury n = 52 (17 in traumatic, 88% alive, and 35 in non-traumatic SCI 77% alive) of which 83% occurred out of the Piedmont Region for a total contribution of 393 person-year.

† Effect size in gender: V = 0.06 in TSCI and V = 0.04 in NTSCI; in age group V = 0.48 in TSCI and V = 0.28 in NTSCI; in injury level V = 0.12 in TSCI and V = 0.03 in NTSCI; in Charlson Comorbidity Index (CCI) V = 0.06 in TSCI and V = 0.14 in NTSCI and in rehabilitation unit V = 0.03 in TSCI and V = 0.1 in NTSCI.

*TSCI*, traumatic spinal cord injury; *NTSCI*, non-traumatic spinal cord injury; *CCI*, combined Charlson Comorbidity Index.

Among individuals with TSCI, 479 were male (80.4%) and 126 were female (19.6%). In the NTSCI group, the 57.4% were male (n = 276) and 42.6% female (n = 189). Women showed slightly higher fatality (27.3%) than men (24.2%), and mortality rates per 1000 person-years were also higher for females (51.1 vs. 40.5), though not statistically significant.

TSCI cases were generally younger, with over 50% under 45 years of age, whereas NTSCI cases were older, with nearly 40% aged 60–74 and over 10% aged 75 or above. The highest fatality rate was observed in individuals over 75 years (55.9%).

Paraplegia was more frequent overall (60.1%), particularly in NTSCI (67.3%), while tetraplegia was more common in TSCI (40.7%). In 52 cases (393 person-years), injury level was undetermined, mostly in NTSCI (n = 35). The strength of association was overall weak (V < 0.3) except for age in the TSCI group (V = 0.48) and NTSCI (V = 0.28).

Mortality rate was higher among individuals with tetraplegia (50.0 per 1000 person-years) compared to those with paraplegia (42.0 per 1000 person-years), although differences varied by etiology.

Most individuals had a CCI score of zero (TSCI0 = 484; NTSCI = 283). However, those with a CCI score ≥3 showed substantially higher mortality, particularly in the NTSCI group (103.8 per 1000 person-years vs. 37.0 in those with CCI = 0).

Over half of the cohort was treated at the Turin SCI unit (n = 564, 52.7%). About 10% received rehabilitation outside Piedmont, showing lower mortality (15.5 per 1000 person-years), and more missing injury-level data, although results were unchanged in sensitivity analyses.

**Fig. 1:**
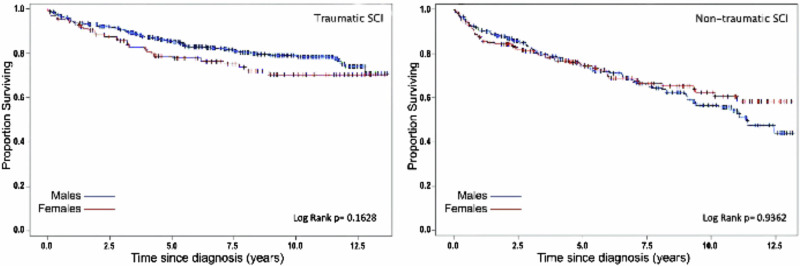
Kaplan-Meier survival plots for spinal cord injury (SCI) in individuals with traumatic SCI and non-traumatic SCI by gender; the blue line represents males, the red line females; (+) censoring times. Figures report P-values for significant differences in survival rates tested using a log-rank test.

**Fig. 2:**
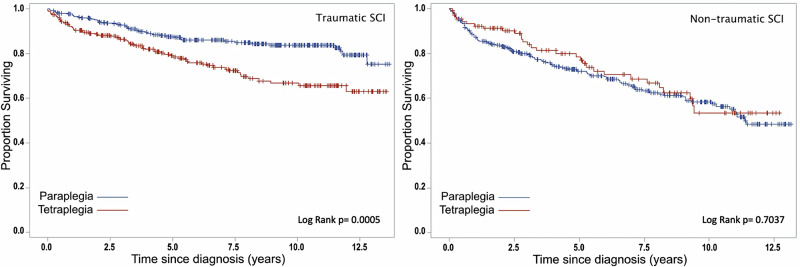
Kaplan-Meier survival plots for spinal cord injury (SCI) in individuals with traumatic SCI and non-traumatic SCI by etiology; the blue line represents individuals with paraplegia, the red line individuals with tetraplegia; (+) censoring times. Figures report P-values for significant differences in survival rates tested using a log-rank test.

Kaplan-Meier analysis showed a non-significant trend toward higher mortality in females compared to males with TSCI across the study period. However, among individuals with NTSCI, females showed a slightly lower likelihood of survival after the eighth year of follow-up (Fig. 1). Individuals with paraplegia had a significantly higher survival probability than those with tetraplegia in the TSCI group (Fig. 2). In contrast, no significant differences in survival time by injury level were observed in individuals with NTSCI.

Multivariate Cox models (Table 2) showed no significant differences in mortality risk between males and females in either the TSCI or NTSCI groups. The hazard ratio (HR) for mortality in women compared to men was 1.05 (95% CI 0.9 to1.3) for TSCI, and 0.94 (95% CI 0.8 to 1.1) for NTSCI, suggesting no sex-based differences in long-term survival outcomes. Adjustment for rehabilitation unit did not change estimates for sex, injury level, or comorbidity. Similarly, mortality risk did not differ between individuals with tetraplegia and those with paraplegia in both TSCI (HR of 1.05, 95% CI 0.9 to 1.3) and NTSCI (HR of 0.95, 95% CI 0.8 to 1.2).

**Table 2:** Hazard Ratios (HRs) with 95% and Confidence Intervals (95% CI) in mortality for people with SCI by gender and level of injury and combined CCI, between 2008 and 2020, Piedmont, Italy.

	TSCI		NTSCI	
	HR^a^	95% CI	HR^a^	95% CI
**Gender**				
Males	1		1	
Females	1.05	0.86–1.29	0.94	0.78–1.14
**Level of injury**				
Paraplegia	1		1	
Tetraplegia	1.10	0.94–1.30	0.95	0.76–1.18
**Combined CCI**				
0	1		1	
1 - 2	1.06	0.84–1.34	1.06	0.84–1.33
≥3	1.81	1.29–2.53	1.24	0.97–1.60

^a^HRs were controlled by Sex, Charlson Comorbidity Index and rehabilitation center.

*CCI* Charlson comorbidity index; *TSCI* Traumatic spinal cord injury; *NTSC* Non-traumatic spinal cord injury.

Individuals with TSCI and a CCI score ≥3, has a significantly higher mortality risk (HR of 1.81, 95% CI 1.3 to 2.5), indicating an 81% increase compared to those with lower CCI scores. A similar but non-significant trend was observed among individuals with NTSCI and a CCI score ≥3 (HR of 1.24, 95% CI 0.8 to 2.2).

At one-year post rehabilitation admission, survival rates were 94% for both males and females with TSCI. However, at five years post-injury, survival was slightly lower in females with TSCI (79%) compared to males (84%), and this trend persisted at 10 years post-injury (70% vs. 79%). Among NTSCI cases, the one-year survival rate after rehabilitation admission was 90% for males and 88% for females, while five-year survival rates were similar between the two sexes (74% for males vs. 75% for females). In TSCI, individuals with tetraplegia had lower survival rates at one year (92% vs. 97%), five years (79% vs. 87%), and 10 years (67% vs. 84%) compared to those with paraplegia. Conversely, in NTSCI, paraplegia had lower survival than tetraplegia at one year (87% vs. 92%) and five years (72% vs. 75%).

### Causes of death

Up to 2018, 173 deaths were recorded (Table 3), representing approximately 90% of all registered deaths in TSCI (n = 69) and 68% of all deaths in NTSCI (n = 104). Within the first 12 months after SCI rehabilitation, main causes among individuals with TSCI were nervous system (21.7%) and circulatory system diseases (17.4%). In contrast, among individuals with NTSCI, the leading causes of death were cancer (40.5%) and circulatory system diseases (21.6%).

**Table 3:** Percentage and absolute number of causes of death within and after 12 months after admission to SCI rehabilitation units for traumatic and non-traumatic SCI between 2008 and 2018 in Piedmont, Italy.

Cause of death (ICD10)	TSCI		NTSCI	
	0 to 12 months	12 months and over	0 to 12 months	12 months and over
	% (n)	% (n)	% (n)	% (n)
Infectious diseases (A00-B99)	8,7 (2)	4,35 (2)	13,51 (5)	17,91 (12)
Cancer (C00-D48)	8,7 (2)	21,74 (10)	40,54 (15)	32,84 (22)
Endocrine, nutritional, and metabolic diseases (E00-E90)	0 (0)	4,35 (2)	–	5,97 (4)
Diseases of the nervous system (G00-G99)	21,74 (5)	15,22 (7)	8,11 (3)	1,49 (1)
Diseases of the circulatory system (I00-I99)	17,39 (4)	19,57 (9)	21,62 (8)	20,9 (14)
Diseases of the respiratory system (incl. influenza and pneumonia) (J00-J99)	–	13,04 (6)	–	–
Diseases of the digestive system (K00-K93)	0 (0)	10,87 (5)	2,7 (1)	10,45 (7)
Diseases of the genitourinary system (N00-N99)	4,35 (1)	4,35 (2)	5,41 (2)	2,99 (2)
Unintentional injuries (V01-X59)	–	4,35 (2)	–	–
Suicide (X60-X84)	–	–	–	1,49 (1)
Other Causes and Unknown^a^	–	–	8,11 (3)	5,97 (4)
Total	100 (23)	100 (46)	100 (37)	100 (67)

Causes of death were available up to 2018, for 173 events.

^a^ICD10: D50-D89, F00-F99, H00-H95, L00-L99, M00-M99, O00-O99, P00-P96, Q00-Q99, R00-R99, X85-Y90.

*TSCI* Traumatic spinal cord injury; *NTSC* Non-traumatic spinal cord injury.

After the first year after admission, NTSCI showed a similar pattern, with slight increases in infectious (17.9%) and digestive system diseases (10.4%). In TSCI, main causes of death shifted, with cancer (21.7%), circulatory system (19.6%), nervous system (15.2%), and respiratory system diseases (13.0%) becoming the most frequently reported.

## Discussion

This study provides a comprehensive assessment of long-term survival, mortality risk factors, and causes of death among individuals with TSCI and NTSCI in a large 12-year population based Italian cohort. The findings highlight age and comorbidities as the strongest predictors of mortality, while sex and injury level (paraplegia vs. tetraplegia) did not significantly influence long-term survival outcomes.

In line with the evolving epidemiology of SCI, our cohort showed an older age at injury onset, particularly for NTSCI, reflecting longer life expectancy, higher prevalence of chronic diseases, and fewer high-energy traumas. These patterns mirror European data reporting a mean onset ages of 55–62 years and increasing comorbidity among NTSCI cases [20–24]. Compared with earlier Italian studies, our findings confirm this demographic transition, with NTSCI predominantly affecting individuals aged 60–74. [23, 24].

Sex and injury level showed limited prognostic relevance, with comparable 10-year survival rates between males and females in both TSCI and NTSCI, although some difference emerged in late follow-up. These findings align with Danish data reporting no survival difference between paraplegia and tetraplegia [25], but differ from Norwegian reports of poorer long-term survival in females with TSCI [13]. Consistent with prior studies, individuals with TSCI and a CCI score ≥3 exhibited a significantly increased mortality risk (HR 1.81), reinforcing the role of frailty and multimorbidity, rather than injury severity, in shaping long-term outcomes [9, 10, 12]. Our results align with Swiss data, where median survival ranged from 1.7 years for malignant etiology, to over 10 years for non-malignant causes [8].

Our findings are consistent with those of other international cohorts. Shavelle et al. reported similar relative life expectancy among SCI populations in the US and UK, despite different absolute mortality rates, highlighting the impact of both systemic and demographic factors [26]. Similarly, a 50-year Australian study found reduced survival in individuals with complete tetraplegia, particularly when combined with age and comorbidity burden [27]. Although based on earlier data, these studies support the relevance of our findings in relation to broader international trends. Circulatory diseases and cancer were the leading causes of death in both TSCI and NTSCI, mirroring trends in the general Italian population [12, 17]. Respiratory diseases were also common in TSCI, especially during later follow-up. While cause-of-death data were available only up to 2018, their stability over time suggests consistent trends. Nonetheless, this limitation warrants caution in interpreting the most recent cause-specific mortality dynamics [17, 22].

### Clinical and public health implications

Our findings suggest that frailty, rather than SCI itself, represents the primary determinant of survival and healthcare needs in in the Italian SCI population. Individuals with TSCI and higher CCI scores showed an increased mortality risk, underscoring the clinical relevance of assessing comorbidity burden. No between-centre mortality differences emerged, including for patients treated outside Piedmont, consistent with standardized national rehabilitation pathways, although some organizational heterogeneity cannot be completely excluded. The observed comorbidity effect reflects pre-injury frailty, as the age-adjusted CCI was computed at baseline from hospitalizations in the prior 12 months, rather than from post-injury complications.

Our results support the regular implementation of frailty screening at the beginning of the rehabilitation process to identify individuals at high risk and enable early, personalized interventions. Rehabilitation plans should extend beyond neurological status to address pre-existing comorbidities and functional limitations. These approaches align with international recommendations for integrated, patient-centered rehabilitation care [28, 29]. These upstream service configurations, particularly in NTSCI, may influence access to specialized rehabilitation and subsequent outcomes. Future linkage with oncology and palliative datasets, combined with multilevel analyses, could help quantify center- and region-level effects. Results also highlight the importance of regional healthcare resource distribution, as seen in other European countries, older NTSCI patients with complex needs may be referred to non-specialized pathways (e.g., oncology, cardiology), which could impact long-term survival outcomes [20]. Integrated oncological and palliative care approaches may represent a promising approach, especially for NTSCI cases with cancer-related etiologies [27]. Furthermore, unmeasured regional factors, such as family support (e.g., caregiver availability), the degree of oncology-palliative integration, and regional policy or budgeting priorities, could influence rehabilitation access, continuity of care, and long-term outcomes.

At the policy level, the demographic and clinical shift in SCI epidemiology requires a reassessment of rehabilitation resource allocation. The growing prevalence of older individuals with NTSCI and multimorbidity increases the complexity of care and highlights the need for a national SCI registry in Italy. Such a registry would support systematic collection of clinical, functional, and sociodemographic data, currently absent in administrative databases, and strengthen health system planning, benchmarking, and research. In addition, Italian mortality patterns show clear socioeconomic gradients and area-level heterogeneity, particularly for circulatory diseases and certain cancers. As individual and area-level socioeconomic data were unavailable, these factors could not be directly assessed but should be considered in judging external validity [30].

### Strengths and limitations

A major strength of this study is the use of a large, population-based cohort with long-term follow-up. The integration of hospital discharge records, mortality databases, and comorbidity indices enabled a robust assessment of survival determinants. Stratified analyses by injury type, sex, injury level, and comorbidity burden provided nuanced insights applicable to both clinical and policy settings. Given the structure of the Italian national health system and the consistency with international data, our findings offer broad generalizability.

Several limitations should be acknowledged.

First, only individuals admitted to rehabilitation were included; thus, deaths during the acute phase were not captured, potentially underestimating early mortality. As survival was measured from rehabilitation admission, variability in prehospital transport, acute transfer, and time-to-center primarily affects selection into the cohort rather than within-cohort survival estimates and may limit generalizability to settings with different pre-admission pathways.

Second, cause-of-death data were available only until 2018, restricting interpretation of recent mortality patterns.

Third, comorbidities were assessed using the combined Charlson Comorbidity Index, calculated from hospitalizations in the year prior to rehabilitation. Although this provides a standardized measure of pre-injury frailty, it is not specific to SCI and excludes key secondary complications (e.g., neurogenic bladder, pressure ulcers) that emerge over time. Only hospital-treated comorbidities were included, and the index was calculated at baseline, limiting its ability to capture evolving health complexity. Reliance on hospital-coded data may also underestimate mild or outpatient condition, likely biasing comorbidity effects toward the null and making observed associations conservative.

Fourth, age was embedded in the CCI scoring, precluding its inclusion as a separate variable and reducing model flexibility. Fifth, the subgroup with high comorbidity burden (CCI ≥ 3) was relatively small, especially among TSCI cases, potentially limiting power and widening confidence intervals. Although models included fixed effects for rehabilitation unit, residual confounding by unmeasured center characteristics (e.g., staffing ratios, multidisciplinary expertise, access to technologies) may persist.

Sixth, limitations in administrative data accuracy may affect diagnostic coding, although SCI diagnoses were established prior to rehabilitation, reducing the risk of misclassification.

Seventh, administrative data lacked information on neurological severity (e.g., AIS grade), detailed injury-level stratifications (e.g., C1–C4 vs. C5–C8), lifestyle factors, and socioeconomic indicators, which are known to influence outcomes. The absence of individual and contextual socioeconomic data (e.g., education, area-level deprivation) prevented analysis of known mortality inequalities in Italy, suggesting potential residual confounding and reinforcing the need for more integrated, multidimensional datasets [30]. While these factors may limit external validity, the overall consistency of our findings with international cohorts supports their transferability to similar contexts. Nevertheless, regional variations in family support, oncology-palliative integration, and budget allocation should be acknowledged as potential modifiers of outcomes and considered when interpreting generalisability.

Future studies should integrate administrative datasets with clinical registries or patient-reported outcome systems, where allowed under privacy regulations. While data protection remains a constraint, such linkage would allow for the inclusion of neurological severity, secondary complications, functional outcomes, and social determinants of health, which are essential to fully understanding survival trajectories and to guiding personalized rehabilitation in SCI populations.

## Conclusion

This population-based study confirms that age, pre-existing comorbidities, and injury type are the strongest predictors of long-term survival in individuals with TSCI and NTSCI, underscoring the central role of frailty and multimorbidity in shaping post-injury outcomes. In contrast, sex and neurological level of injury showed limited prognostic relevance after adjustment. As SCI increasingly affects older individuals with complex health profiles, mortality patterns mirror those of the general population, with cancer and circulatory diseases emerging as the predominant causes of death. Overall, survival reflects the combined influence of injury etiology, underlying health conditions, and aging-related frailty.

Rehabilitation strategies should therefore prioritize early frailty screening, proactive comorbidity management, and the personalization of care pathways to improve long-term outcomes. To support these goals, the creation of a national SCI registry in Italy is strongly recommended. Such a registry would enable comprehensive tracking of clinical, functional, and sociodemographic data, informing more precise care planning, research, and health policy development.

## Data Availability

The datasets analyzed in the current study are not publicly available due to GDPR restrictions but are available from the corresponding author on reasonable request.
